# Cavity‐Enhanced Eye‐Readable Detection of 0.1% H_2_ via Active Modulation of Charge‐Transfer Plasmons

**DOI:** 10.1002/advs.77338

**Published:** 2026-08-27

**Authors:** Xuemin Zhang, Shunsheng Ye, Lubing Cai, Chao Li, Zheng Cai, Jin Zhang, Fei Liu, Tieqiang Wang, Boxin Wei, Hongbo Zeng

**Affiliations:** ^1^ Department of Chemistry College of Sciences Northeastern University Shenyang Liaoning People's Republic of China; ^2^ Institute of Metal Research Chinese Academy of Sciences Shenyang People's Republic of China; ^3^ Department of Chemical and Materials Engineering University of Alberta Edmonton Canada; ^4^ National & Local Joint Engineering Laboratory for High‐Performance Metal Structure Material and Advanced Manufacturing Technology College of Materials and Metallurgy Guizhou University Guiyang Guizhou People's Republic of China; ^5^ School of Mechanical and Aerospace Engineering Nanyang Technological University Singapore Singapore

**Keywords:** angle/background‐insensitive color display, eye‐readable hydrogen sensor, hydrogen‐induced charge transfer, plasmonic cavity, rapid response

## Abstract

Eye‐readable hydrogen sensors (EHSs) are essential for the safe deployment of hydrogen energy technologies, owing to their intuitive optical readout capabilities. Nevertheless, current plasmonic EHS designs are hindered by high critical discoloration concentrations (CDCs), slow response times, and inconsistent discoloration behavior. Herein, we present a Fabry‐Pérot cavity‐type EHS composed of close‐packed Au@Pd@oxide core‐shell nanoparticles that operates via a hydrogen‐induced charge‐transfer (HICT) mechanism. Hydrogen exposure reduces of the oxide shell of Au@Pd@oxide and forms Au@Pd, transforming the plasmonic resonance from a gap mode to a charge‐transfer mode, resulting in a vivid, reliable color change. This design achieves an ultra‐low CDC of 0.1% H_2_, a rapid response time of 4 s, and robust performance across diverse environmental conditions, surpassing U.S. Department of Energy safety targets. This work establishes HICT as a novel strategy for active plasmon modulation and highlights cavity‐engineered plasmonic architectures as promising platforms for next‐generation hydrogen safety sensors.

## Graphical abstract

A cavity‐structured nanoparticle sensor is developed for highly sensitive, eye‐readable hydrogen detection. Its excellent colorimetric response arises from a hydrogen‐induced charge‐transfer mechanism: reduction of the dielectric oxide shell in H_2_ triggers a transition from the plasmonic gap mode to the charge‐transfer plasmon mode. The cavity architecture further enables reliable sensing that is largely independent of the substrate, viewing angle, and interfering gases.

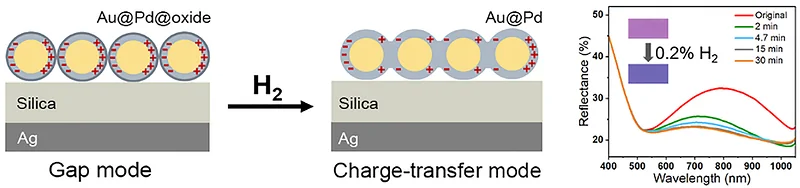

## 1 Introduction

Hydrogen gas is a promising clean energy carrier with broad potential applications in industry, transportation, and residential applications. However, its colorless, odorless, and highly flammable nature, coupled with a low ignition threshold and broad explosive range in air (4–75 vol%), pose serious safety risks. Real‐time and reliable hydrogen leak detection is thus critical to prevent catastrophic accidents during production, storage, transport, refueling, and utilization of hydrogen [1, 2, 3]. Among existing sensor technologies, EHSs offer distinct advantages, including intrinsic safety, remote contact‐free readout, near‐zero power consumption, and the potential for low‐cost, large‐area deployment owing to their simple visual detection mechanism [4, 5, 6].

Recently, various type of EHSs based on metal oxides (such as WO_3_), chemical dyes (such as resazurin), and nanoplasmonic systems have been developed [7, 8, 9]. Among them, Pd‐based plasmonic nanostructures represent one of the most classical and extensively investigated approaches for optical hydrogen sensing, owing to the intrinsic hydrogen affinity of Pd and the ability of selective transduction of H_2_–Pd interaction into robust optical readouts even at room temperature [10, 11, 12, 13, 14, 15]. Pd‐based optical hydrogen sensors generally operate via two primary mechanisms: hydrogen‐induced dielectric‐constant change (HIDC) and hydrogen‐induced volume expansion (HIVE). The HIDC mechanism exploits the efficient hydrogen absorption ability of Pd to quantitively detect H_2_ through a spectral shift of the plasmon band. However, the slight difference in dielectric constant between Pd and PdH*_x_* (0 < *x *< 1) is insufficient to trigger perceptible color changes [16, 17]. Alternatively, the HIVE mechanism leverages the huge morphological changes of nanostructures caused by Pd expansion upon hydrogen uptake, which alters the light‐matter interaction and leads to visible color variations. This approach has enabled various designs such as Au@Pd nanorods and nanoparticles (NPs) array [18, 19], Janus Pd/polymer nanofiber array [20], Pd/Cr/Ti nanomembrane micro‐rolls array [21], and suspended Pd film, etc [22]. However, substantial Pd volume expansion occurs only during the *α‐β* phase transition at hydrogen concentrations above 1%. Consequently, HIVE‐based sensors typically exhibit high critical discoloration concentrations (CDCs, the lowest hydrogen concentration to trigger a noticeable color change), limiting their ability to provide early visual warning at the lower end of the 0.1%–10% H_2_ measurement range recommended by the U.S. Department of Energy (DOE) [23]. What is worse, Pd expansion is often constrained by the substrate, resulting in sluggish response times. For example, Au@Pd NP arrays have shown response times ranging from several minutes to tens of minutes depending on structural configuration and sensing conditions [19, 24].

In addition to the aforementioned limitations, plasmonic structural colors are susceptible to the background color, observation mode (transmission or reflection), and viewing angle. This remains a widely recognized yet unresolved problem, potentially leading to uncertainty, unreliability, and even the risk of false alarms in practical applications [25, 26, 27]. An ideal EHS must thus simultaneously achieve a low CDC, rapid response, broad optical modulation, and angel/background‐insensitive color display. However, to our best knowledge, no such plasmonic EHS has ever been reported yet.

Herein, a hydrogen‐induced charge‐transfer (HICT) mechanism is developed to actively modulate plasmonic modes for ultrasensitive and reliable detection of hydrogen by naked eye. The HICT mechanism is realized in a close‐packed array of Au@Pd@oxide core‐shell NPs prepared via a three‐phase interfacial self‐assembly. The ultrathin native oxide shell on Pd functions as a dielectric layer that enables strong interparticle coupling (gap mode). Upon H_2_ exposure, rapid reduction of the oxide shells switches the gap mode to charge transfer (CT) mode, triggering a reflectance drop and visible color shift. To enhance sensing reliability, we integrate the Au@Pd@oxide NP array with a silica spacer and a Ag mirror, forming a Fabry‐Pérot (FP)‐type cavity. This configuration eliminates background/angle dependence via the Ag mirror and suppresses optical noise through broadband absorption. Consequently, the HICT‐based H_2_ sensor demonstrates a rapid color change from reddish violet to blue within 4 s when exposed to 0.2%–100% H_2_/air mixtures. A perceptible color change can be achieved even in 0.1% H_2_. As proof of concept, flexible tape‐supported sensors demonstrate perfect compatibility with curved surfaces of gas pipelines, showing great potential in visual leak localization in real applications. As far as we know, this work represents the first experimental and theoretical demonstration of plasmonic mode switching from gap mode to CT mode for hydrogen sensing, offering a scalable and robust platform for next‐generation eye‐readable sensors for real‐world visual leak detection.

## 2 Results and Discussions

### 2.1 Morphological and Chemical Characterization of the HICT‐Based Sensor

**FIGURE 1:**
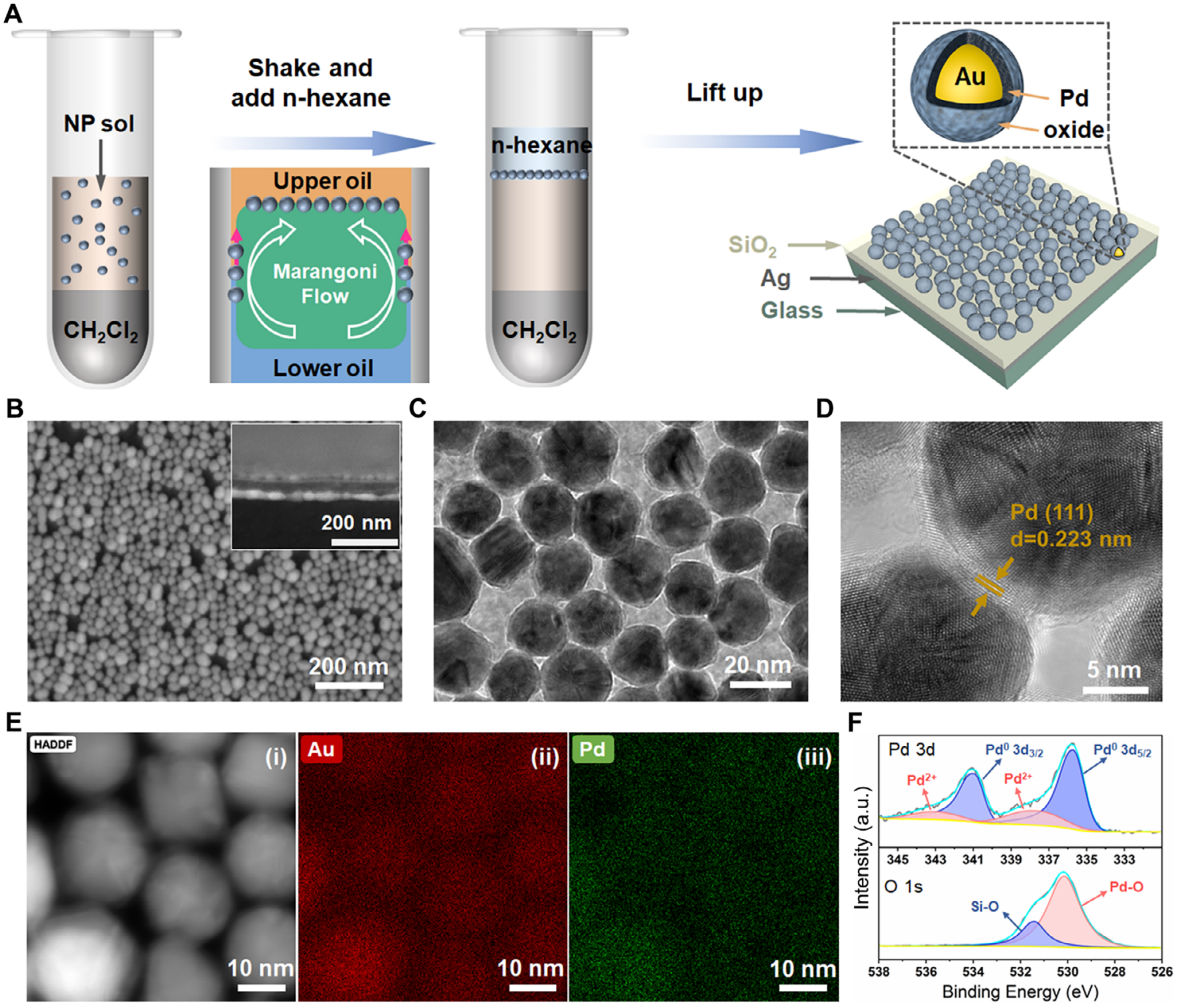
Preparation and characterization of the HICT‐based sensor. (A) Schematic diagram for the assembly of close‐packed Au@Pd@oxide NPs and preparation of HICT‐based sensor with a FP cavity structure. (B) SEM image of the top Au@Pd@oxide (19/2) NP array. Inset is the cross‐section view of the cavity structure. (C, D) TEM images of closed‐packed Au@Pd@oxide (19/2) NPs. (E) Enlarged STEM image of the Au@Pd@oxide (19/2) NP array (i) and EDX elemental distribution of Au (ii) and Pd (iii). (F) XPS spectra of the pristine Au@Pd@oxide (19/2) NPs.

The sensor architecture comprises a FP cavity with three functional layers: a top monolayer of close‐packed Au@Pd@oxide NPs, a silica interlayer deposited via spin‐coating, and a bottom Ag mirror. Experimentally, the close‐packed NP monolayer was fabricated using three‐phase interfacial self‐assembly driven by Marangoni compression [28], where surface tension gradients between the water/n‐hexane (upper) and water/dichloromethane (lower) interfaces induce monolayer compression at the water/n‐hexane interface. Then, the close‐packed NP monolayer at the interface was transferred onto the surface of Ag/silica layer, obtaining the FP cavity sensor (Figure 1A).

Top‐view and cross‐sectional scanning electron microscopy (SEM) images in Figure 1B confirm the well‐defined trilayer structure. Transmission electron microscopy (TEM) reveals short‐range ordered yet close‐packed Au@Pd@oxide NPs with negligible interparticle separation (Figure 1C,D) [28]. High‐resolution TEM resolves the core‐shell configuration, showing a characteristic shell lattice spacing of 0.223 nm corresponding to the Pd (111) plane (Figure 1D) [29]. Elemental mapping by high‐angle annular dark field‐scanning TEM‐energy dispersive x‐ray (HAADF‐STEM‐EDX) verifies Au localization in the NP core and Pd enrichment at the shell surface(Figure 1E). The x‐ray diffraction (XRD) peaks of obtained NPs located at 38.8°, 44.1°, 65.1°, and 78.2° can be assigned to the (111), (200), (220), and (311) crystal planes of metallic Au, respectively (Figure S1). These peaks are slightly shifted toward higher angles compared with standard Au diffraction peaks, indicating the presence of Pd in the samples. According to previous work, there is always a native oxide layer on the surface of metallic Pd [30]. Although this oxide layer is challenging to distinguish via TEM, its presence can be proved by the x‐ray photoelectron spectroscopy (XPS) measurement. As shown in Figure 1F, the Pd 3d spectrum exhibits signatures of metallic Pd0 (335.8 and 341.0 eV) [31], alongside distinct Pd^2+^ states (339.0 and 344.2 eV). Complementary O 1s peaks at ∼530 eV (Pd─O lattice oxygen) and ∼532 eV (Si─O from substrate) further substantiate the presence of the oxide shell [31].

For clarity in subsequent discussions, the core‐shell NPs are designated as Au@Pd@oxide (x/y), where x and y represent the Au core diameter and total Pd‐based shell thickness (including the oxide layer), respectively. For instance, NPs in Figure 1D, featuring a 19 *± *2 nm core and 2 ± 0.5 nm shell, are denoted Au@Pd@oxide (19/2).

### 2.2 Sensing Performance of the HICT‐Based EHS

**FIGURE 2:**
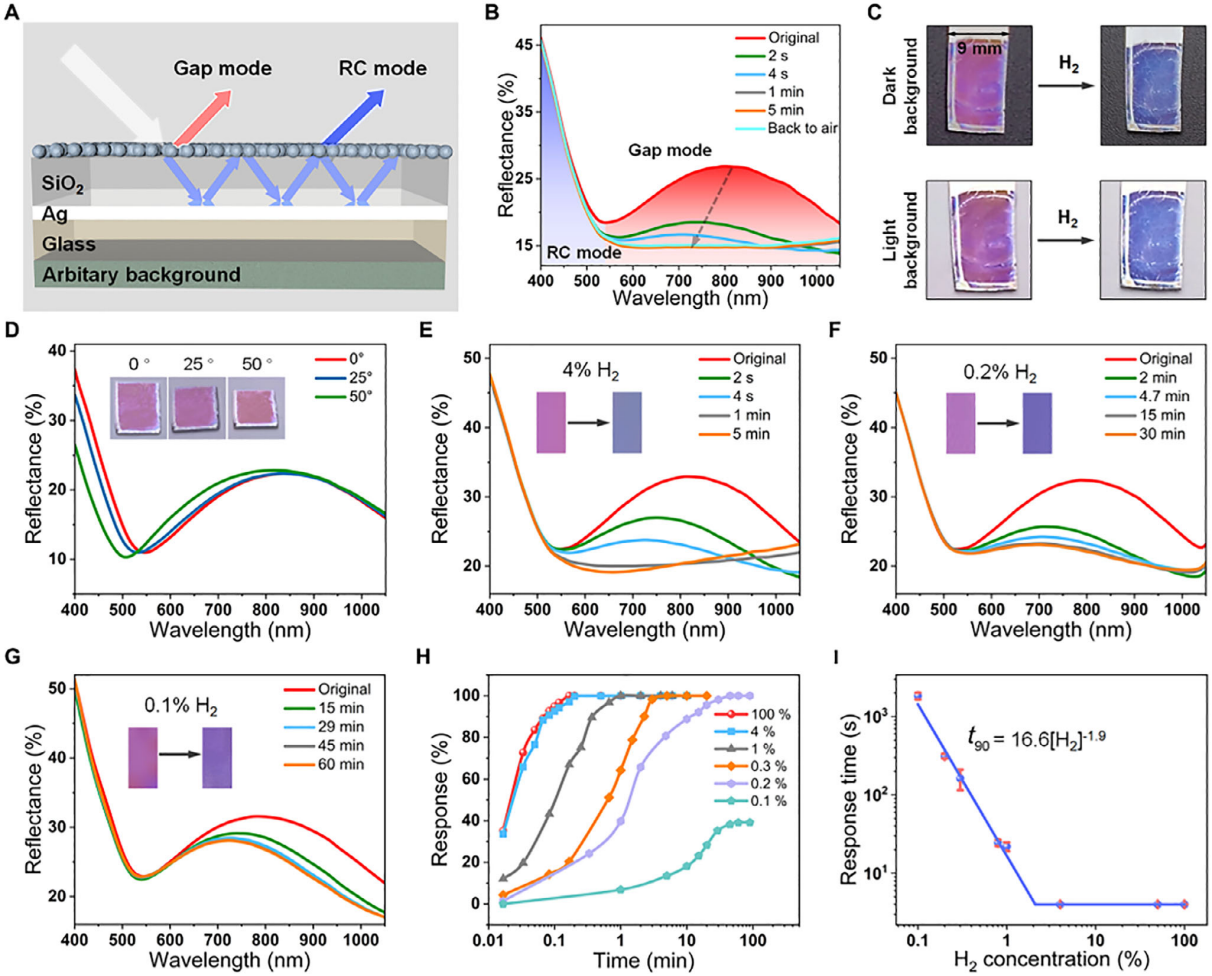
Sensing performance of the HICT‐based sensor. (A) Schematic diagram of the interaction between incident white light and the as‐prepared sensor on an arbitrary substrate. (B) Real‐time change of the reflection spectra of the HICT‐based sensor upon exposure to pure H_2_. (C) Hydrogen‐induced discoloration of the HICT‐based sensor against dark and light backgrounds. (D) Reflection spectra of the sensor measured at different angles. Inset shows the photos of the sensor taken at corresponding angles. (E–G) Real‐time changes of the reflection spectra when HICT‐based sensors were exposed to 4% (E), 0.2% (F), and 0.1% (G) H_2_ (with air as background). (H) Responses of sensors at different H_2_ concentrations as functions of time. (I) Response time (*t*_90_) as a function of H_2_ concentration.

For FP cavity structure incorporating a disordered plasmonic top layer, it exhibits a broad absorption with selective reflection peaks. According to the coupled mode theory [32, 33, 34], the coupling efficiency (*η*) for the k^th^ cavity mode is given by:

$$\eta =\frac{2\gamma_{e}/\gamma_{k0}}{{\left(1+\gamma_{e}/\gamma_{k0}\right)}^{2}}$$

where γ_e_ and γ_k0_ represent the coupling rate and intrinsic decay rate, respectively. Optimal absorption occurs when γ_e_ ≈ γ_k0_. The disordered nanoparticle arrangement induces convergence of γ_k0_, enabling broadband absorption across most wavelengths (light blue arrows, Figure 2A). At wavelengths where γ_e_ and γ_k0_ are mismatched, energy escapes the cavity, generating selective reflective‐cavity (RC) modes (blue arrows, Figure 2A).

Reflection spectra of the HICT‐based sensor (58‐nm silica layer) reveal two distinct features (Figure 2B): an RC mode below 520 nm and a plasmonic gap mode (520–1000 nm) from the Au@Pd@oxide NPs. These collectively generate a reddish‐violet color. Upon H_2_ exposure, the gap mode undergoes rapid (<4 s), irreversible attenuation accompanied by a blue shift, while the RC mode remains stable. This transitions the apparent color from reddish‐violet to blue (Video S1). Crucially, this discoloration is substrate‐insensitive against both light and dark backgrounds (Figure 2C). Angular dependence is a common characteristic of plasmonic structural colors; however, in our sensor design, this issue is resolved by leveraging the short‐range ordered arrangement of the top NP array [27, 35]. As shown in Figure 2D, the optical property and apparent color of the sensor are nearly unchanged from different viewing angles (0°–50°). Sensors with varying NP structures were also evaluated (Figure S2). The control experiments show that sensors employing only Au NPs or only Pd NPs as the top sensing layer exhibit no obvious color change upon exposure to pure H_2_, confirming that Au@Pd@oxide core‐shell structure is essential. For core‐shell NPs, increasing Au core diameter redshifts the gap mode beyond the visible range, while thicker Pd shells dampen the plasmon due to Pd's high imaginary permittivity [36], both of which diminish visible color changes. Thus, ​Au@Pd@oxide (19/2)​​ NPs were selected for optimized visible response in the following experiments and calculations unless otherwise specified​.

Sensor response (*S*) is defined as (*I*_a_—*I*_g_)/*I*_a_ × 100%, where *I*_a_ and *I*_g_ denote gap‐mode intensities in air and H_2_, respectively (Figure S3). Across 0.2%–100% H_2_, the sensor achieves an on‐off modulation of the gap mode (*S* = 100%), along with a consistent color transition from reddish violet to blue (Figure 2E,F). Even at 0.1% H_2_, a discernible color change (*S* = 40%) occurs (Figure 2G). Additionally, the color difference (Δ*E*) of the HICT‐based EHS was quantified using the CIELAB color space [8]. The fully developed color change (*S* = 100%) gives a ΔE of ∼31, while 0.1% H_2_ exposure (*S* = 40%) produces a ΔE of ∼18, far exceeding the Δ*E* = 5 threshold for naked‐eye distinguishability. The response time (*t*_90_), defined as the time reach to 90% maximum response, follows an inverse relationship with H_2_ concentration (Figure 2H), scaling as [H_2_]^─1.9^ between 0.1% and 2% and plateauing at ∼4 s for concentrations ≥ 4% (Figure 2I).

**FIGURE 3:**
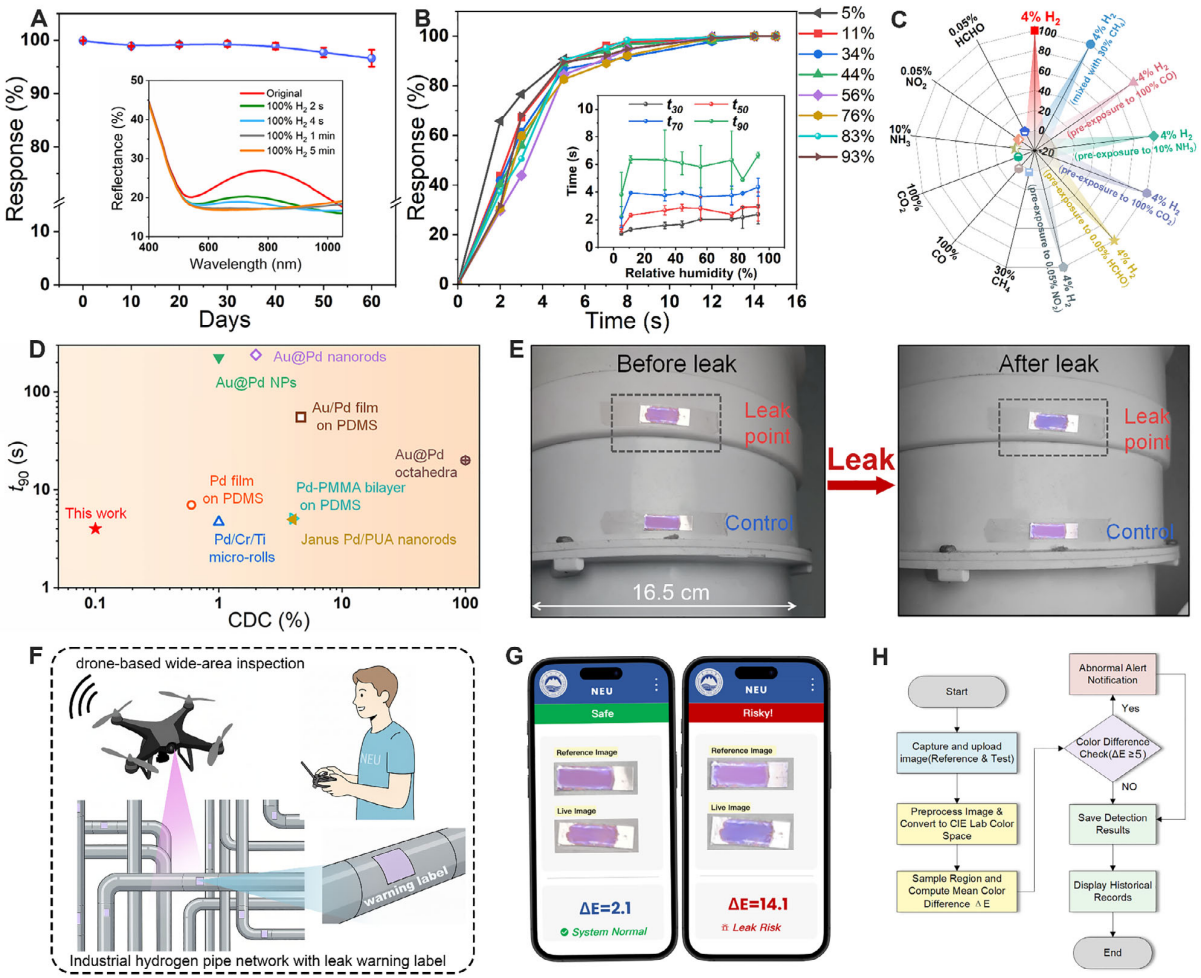
Sensing performance of HICT‐based sensors in practical situations. (A) Long‐term stability of the HICT‐based sensor. Inset is the performance of sensor stored for 2 months. (B) Real‐time response of the HICT‐based sensor in H_2_ environment with different RH. Inset shows the response kinetics at different RH. (C) Selectivity of the HICT‐based sensor. (D) Sensing performance of state‐of‐the‐art plasmonic EHSs in terms of response time (at 4% H_2_) and CDC. Open and solid symbols indicate that H_2_ was diluted with N_2_ and air, respectively. Au@Pd octahedra‐based EHS operated in water. (E) Flexible HICT‐based EHS serves as a warning label on the curved surface of an H_2_ gas pipeline. The upper label is close to the leak point, and the lower one is the control group. (F) Schematic illustration of drone‐assisted, wide‐area inspection using HICT‐based eye‐readable warning labels for remote hydrogen leak visualization. (G) Screenshots of the custom‐developed smartphone application for on‐site risk assessment. (H) Flowchart of the detection algorithm.

For practical deployment, we further investigated the reproducibility, long‐term stability, humidity resistance, and selectivity. To assess reproducibility and long‐term stability, multiple samples were fabricated simultaneously and stored under ambient conditions without any special treatment. After predetermined storage periods, individual sensors were tested under 4% H_2_. The response performance of sensors stored for different durations was then compared to evaluate the reproducibility and long‐term stability of the device. The small variations in response, represented by the error bars in Figure 3A, confirm the excellent reproducibility of the sensors, indicating that structural imperfections such as voids and cracks in the NP arrays have negligible influence on performance. As shown in Figure 3A, the devices maintained nearly 100% of their initial response after ∼2 months, and no obvious variation in response kinetics was observed, demonstrating the robustness of the ultrathin native oxide shell and the good long‐term stability of the HICT‐based EHSs. As shown in Figure 3B, the response to 4% H_2_ remains unaffected across relative humidity levels of 5%–93%. To provide a more comprehensive evaluation of the response kinetics, we further analyzed the characteristic response times required to reach 30%, 50%, 70%, and 90% of the maximum response (denoted as *t*_30_, *t*_50_, *t*_70_, and *t*_90_, respectively). The results indicate that the sensor exhibits a slightly faster response at 5% RH, whereas the response kinetics are nearly identical over the remaining humidity range. Owing to the specific interactions between H_2_ and Pd/PdO, our EHS possesses extremely high selectivity toward H_2_. In particular, it shows 2–3 orders of magnitude stronger response to 4% H_2_ than to interferents, including NO_2_, CO, and CH_4_ (Figure 3C and Figure S4). Furthermore, the sensor maintains its original hydrogen response in mixed gas environments (e.g., 4% H_2_/30% CH_4_) and after exposure to poisoning gases (e.g., pre‐exposure to CO followed by hydrogen detection), demonstrating excellent resistance to interference, poisoning, and deactivation.

Compared to conventional plasmonic H_2_ sensing mechanisms (HIDC and HIVE), HICT offers transformative advantages (Figure 3D and Table S1). HIDC‐based sensors fail to achieve visible color changes despite complex nanostructuring [37, 38, 39]. As far as we know, the only successful example is the Au@Pd octahedra NP sol, which exhibited a spectral shift of ∼22 nm and a color change from purplish red to purple upon exposure to pure H_2_ [40]. However, this liquid system is unsuitable for practical H_2_ detection. Meanwhile, HIVE‐based EHSs suffer from high CDCs (∼1%), slow responses, angle‐dependent readouts, and complex synthesis processes [18, 19, 20, 21, 22, 24]. By contrast, the HICT‐based sensor have achieved rapid response and pushed the CDC down to 0.1% that can meet the strict requirement set by the US DOE. Besides, the color change of this EHS is independent of background, viewing angle, interfering gases, and moisture. Notably, the discoloration of the HICT‐based sensor is irreversible, distinguishing it from all previously reported reversible Pd‐based plasmonic hydrogen sensors. Consequently, the device functions as a permanent visual indicator of hydrogen exposure, enabling retrospective identification of leakage events even after hydrogen has dissipated, such as applications in hydrogen transport pipelines and residential gas systems, where recording leakage history is essential for safety management.

Furthermore, the HICT‐based EHS exhibits a substantially lower CDC and faster response kinetics compared with previously reported non‐plasmonic EHS platforms. To quantitatively evaluate the sensing performance, the coloring rate (*R*_c_) is introduced, which is defined as:

$$R_{c}=\Delta E/\left(t_{90}\times \left[H_{2}\right]\right)$$

where Δ*E* represents the color difference, and *t*_90_ denotes the response time, and [H_2_] corresponds the hydrogen concentration (vol%). *R*_c_ for HICT‐based EHS is ∼193.8 s^−1^, representing a 6–277 fold enhancement over previously reported non‐plasmonic EHS platforms (Table S2 and Figure S5). This result highlights the superior hydrogen‐to‐color transduction efficiency enabled by the HICT mechanism.

As a proof of concept for practical applications, HICT‐based EHSs were fabricated on polyethylene terephthalate (PET) films. The flexible sensors readily conform to moderately curved surfaces, such as gas pipelines (diameter of 16.5 cm), enabling their use as visual hydrogen leak warning labels (Figure 3E). Upon hydrogen exposure, the sensors rapidly change from reddish violet to blue, directly visualizing the leak location while exhibiting comparable sensing performance in both the flat and bent states (Figure S6). This irreversible response provides a critical historical record for post‐incident analysis [41, 42, 43]. Beyond direct visual recognition, the HICT‐based EHSs have the potential to be integrated with low‐cost machine‐vision systems for automated hydrogen leak detection. For example, a smartphone application can be designed to illustrate this future application (Figure 3F,G). The algorithm (Figure 3H) captures a reference image of the sensor, converts it into the CIELAB color space, and stores its color profile. During operation, a newly acquired image is analyzed to calculate the Δ*E*, which is then compared with a preset threshold (Δ*E* = 5 in this work) to generate a “Safe” or “Risky!” alert. Although this preliminary demonstration has not yet been systematically optimized or validated under practical operating conditions, it highlights the potential for integrating HICT‐based EHSs with readily available imaging devices, such as smartphones and drones, for future portable and wide‐area hydrogen leak monitoring.

### 2.3 Sensing Mechanism of the HICT‐Based EHS

**FIGURE 4:**
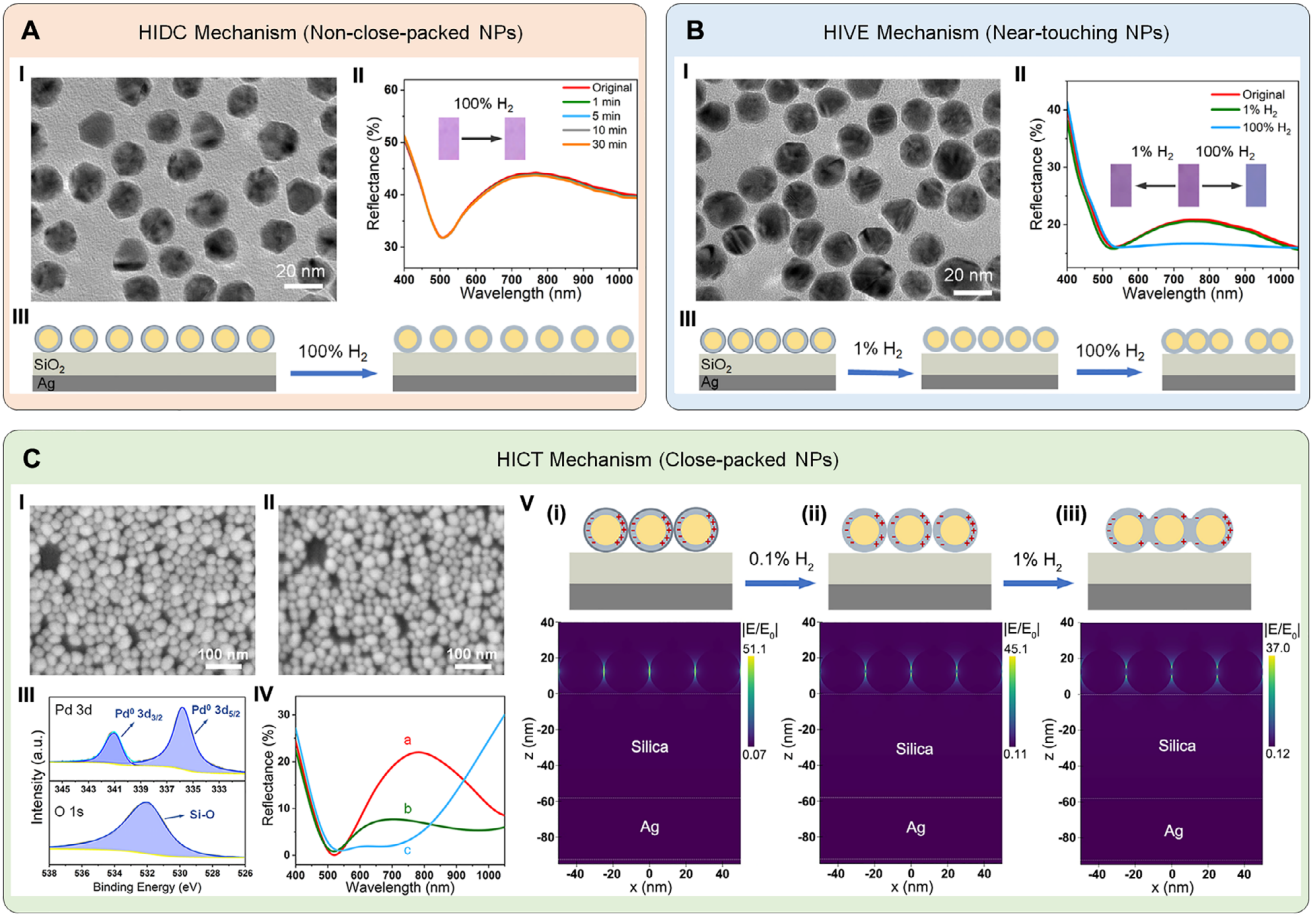
A unified understanding of the sensing mechanisms of Au@Pd@oxide NPs with various structural features. (A) HIDC mechanism for non‐close‐packed NPs. TEM image (A‐I) and sensing performance (A‐II) of the non‐close‐packed Au@Pd@oxide (19/2) NPs stabilized by PVP. (A‐III) Schematic illustration of HIDC sensing mechanism. (B) HIVE mechanism for near‐touching NPs. TEM image (B‐I) and sensing performance (B‐II) of the near‐touching Au@Pd@oxide (19/2) NP array stabilized by sodium citrate and ascorbic acid. (B‐III) Schematic illustration of HIVE sensing mechanism. (C) HICT mechanism for close‐packed NPs. (C‐I, II) SEM images of the close‐packed Au@Pd@oxide (19/2) NPs before (I) and after (II) exposure to 0.1% H_2_. (C‐III) XPS Pd 3d and O 1s spectra of the Au@Pd@oxide (19/2) NPs reduced by 0.2% H_2_. (C‐IV) Simulated reflection spectra of HICT‐based sensors comprising top layers of close‐packed Au@Pd@oxide (19/2) NPs (line a), Au@Pd (19/2) NPs (line b), and slightly fused Au@Pd (19/2) NPs (line c). (C‐V) Schematics of the surface charge distributions of the close‐packed Au@Pd@oxide (19/2) NPs (i), Au@Pd (19/2) NPs (ii), and slightly fused Au@Pd (19/2) NPs (iii). The opposite signs indicate the charge density induced by plasmon resonance. The lower panels are their corresponding near‐field electric field distributions at 780 nm (i), 704 nm (ii), and 624 nm (iii).

Experimental evidences verify close‐packing as the most important structural feature that supports the exceptional sensing performance of Au@Pd@oxide NPs. To investigate this phenomenon systematically, we engineered NPs with controlled interparticle separations using distinct surface ligands and assembly methods. When polyvinyl pyrrolidone (PVP) was used for stabilizing NPs, interparticle distance is 5 ± 2 nm. In this case, the sensor exhibited only a minimal spectral shift (∼5 nm redshift) and an indiscernible color change upon exposure to pure H_2_ (Figure 4A). This response represents the average performance of HIDC‐based plasmonic hydrogen sensors [17, 37, 38, 39]. confirming that the slight change of dielectric constant alone cannot drive significant optical modulation. Using sodium citrate and ascorbic acid as ligands without Marangoni compression yielded near‐touching NPs (1 ± 0.5 nm separation). While these structures showed optical changes in pure H_2_, they failed to respond to 1% H_2_ (Figure 4B). This limitation stems from the HIVE dominance: these near‐touching NPs irreversibly aggregate in pure H_2_ due to the volume expansion of the Pd shells [24]. However, significant expansion of Pd only occurs during the α‐β phase transition in >1% H_2_ and the movement of NPs is constrained by the substrate at the same time. Therefore, near‐touching NPs have a much slower response rate and an elevated CDC [24].

The function of the close‐packed structure was figured out through SEM and XPS characterization and computational modeling. Unlike the near‐touching Au@Pd NPs that the response is induced by NP's aggregation in H_2_, SEM images reveal no structural reorganization in close‐packed NPs after exposure to 0.1% H_2_ (Figure 4C‐I, II), implying HIVE is not the sensing mechanism. XPS confirm complete disappearance of Pd^2+^ signatures and Pd─O bonding (Figure 4C‐III). These results indicate the reduction of Au@Pd@oxide NPs to Au@Pd NPs without morphological disruption. Finite‐difference time‐domain (FDTD) simulations provide further mechanistic insights. The close‐packed Au@Pd@oxide(19/2) NPs exhibit a strong reflection peak (∼780 nm) corresponding to the gap mode (line a, Figure 4C‐IV). Reduction to Au@Pd NPs attenuates and blue‐shifts this peak (line b, Figure 4C‐IV), while fused NPs show complete suppression (line c, Figure 4C‐IV). These calculated reflection spectra and their evolution coincide with our experimental observations. Near‐field mapping at 780 nm reveals intense electric field localization within sub‐1 nm gaps between oxide‐separated NPs (Figure 4C‐V‐i), confirming strong plasmonic coupling mediated by the dielectric oxide spacer [44]. The ultrathin oxide shell enabled charge oscillation while preventing direct electron transfer.

Hydrogen‐induced oxide reduction eliminates the dielectric barrier and transforms Au@Pd@oxide NPs into directly contacted Au@Pd NPs. This establishes metallic junctions that allows for direct CT and an oscillating current flow between adjacent Au@Pd NPs [45, 46]. Consequently, the conductive short‐circuiting of plasmon oscillations leads to dampened gap mode and strengthened CT mode, which redistributes the near‐field electric field and modifies the far‐field spectra. The localized electric field becomes around the junction and the maximum field intensity is lower than that before reduction (Figure 4C‐V‐ii). The weakened plasmon coupling leads to the blue shift and attenuation of the far‐field reflection peak (line b, Figure 4C‐IV). Our experimental and simulated results also agree well with previously reported observations [46, 47]. For example, it was reported that the extinction intensity of Au NP dimers diminished when the gap mode transitioned to CT mode. The band energy and intensity of the weakened gap mode are also very sensitive to the junction profile [45]. According to our computational results, a thicker junction could lead to completely quenched gap mode in far field (line c, Figure 4C‐IV) and further weakened localized electric field around the junction (Figure 4C‐V‐iii). Meanwhile, the enhanced electron transfer between NPs is manifested in the substantially increased reflectance at longer wavelengths (>800 nm). This theoretically predicted structure is experimentally reasonable because the freshly reduced Pd atoms have a relatively high diffusion ability, thus promoting the fusion of adjacent NPs. The fusion of Au@Pd NPs can be partially evidenced by the expanded cracks between NP domains after exposed to high‐concentration H_2_ (Figure S7). Therefore, even the re‐oxidation of the reduced Pd surface under ambient conditions will not restore the original oxide shell structure required for gap‐mode plasmon coupling. This result further supports the applicability of the sensor as a permanent hydrogen exposure indicator.

Based on the above discussion, a unified explanation is reached to understand the distinct sensing performances of previously reported plasmonic hydrogen sensors (Figure 4). An evolution of sensing mechanism from HIDC mechanism to HIVE mechanism and finally to HICT mechanism is observed with the reduction of interparticle distances. Compared with conventional HIDC and HIVE‐based plasmonic hydrogen sensors, HICT‐based sensors exhibit more significant response and much lower CDC as a result of the transition of plasmon modes during the sensing process. Moreover, the HICT mechanism achieves an unprecedented response speed. Generally, the reduction of PdO in H_2_ follows an S‐shaped curve, i.e., a long initial induction period followed by a fast reduction (see also the response curve at 0.1% H_2_ in Figure 2H). According to previous reports, PdO‐based EHSs typically require several minutes to complete the discoloration process (*t*_90_) under 4% H_2_ [41, 42, 43], whereas the response time in our system is only ∼4 s. This acceleration originates from Pd‐catalyzed oxide reduction kinetics: Pd lowers the H_2_ dissociation barrier on PdO (101) surfaces from 0.38 to 0.12 eV, while simultaneously facilitating H diffusion and H_2_O formation [48]. This catalytic synergy between the Au@Pd core‐shell structure and oxide interface bypasses the slow induction period characteristic of bulk PdO reduction. Thus, the HICT mechanism uniquely combines plasmonic mode switching with catalytic enhancement, enabling both large optical modulation and rapid response unattainable through conventional approaches.

### 2.4 Influence of FP Cavity Structure on HICT‐Based EHS Performance

The exceptional sensing performance of our hydrogen sensor arises from the synergistic combination of the HICT mechanism and the optimized FP cavity architecture. To isolate the cavity's contribution, we evaluated a monolayer sensor comprising only a close‐packed Au@Pd@oxide NP array on glass substrate (Figures S8 and S9A). While this configuration retained a low CDC endowed by the HICT mechanism (Figure S8), it exhibited significant limitations: its discoloration response depended critically on background color. The apparent color transitioned from deep blue to gray against a white background but showed negligible change against a dark background (Figure S9B). This inconsistency originates from background‐dependent light interactions, as confirmed by FDTD simulations (Figure S9C). The transmitted light (∼470 nm) would be rescattered or absorbed differently by underlying materials, which has been one of the greatest challenges in plasmonic sensing systems [5, 25, 26, 27]. Moreover, the monolayer‐type sensor failed to achieve full modulation of the plasmon band upon pure H_2_ exposure. The sensor response increases with time in pure H_2_ gas and finally reaches a plateau of S = ∼80% (Figure S9D). In stark contrast, the simple FP cavity configuration eliminates these limitations by providing a spectrally stable, low‐noise background that isolates optical responses to hydrogen‐induced changes in the Au@Pd@oxide NP array [32, 33]. This architecture ensures background invariance while enabling near‐total reflectance modulation.

**FIGURE 5:**
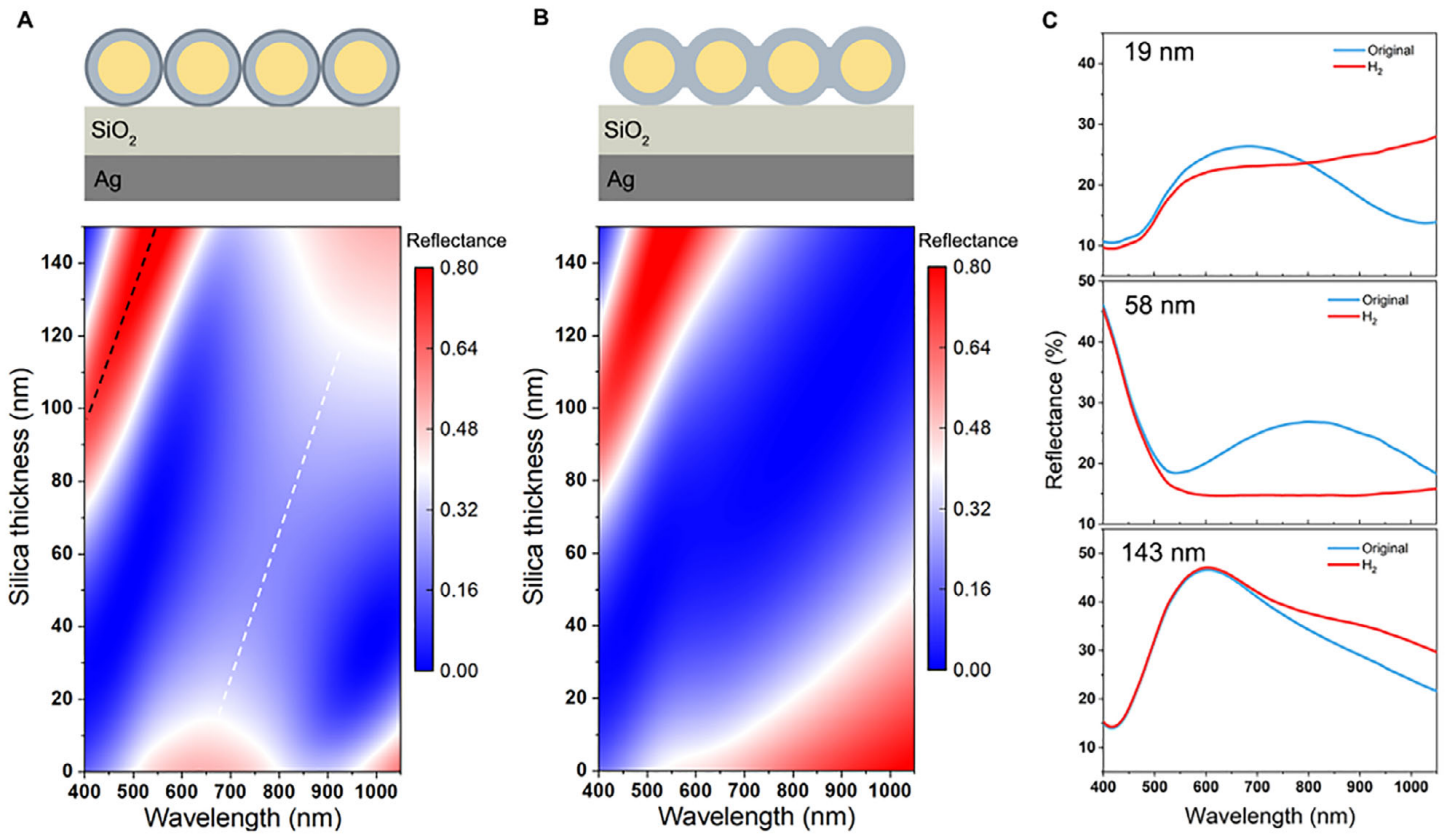
Effect of the silica thickness on sensing performance. (A) Contour plot of simulated reflection spectra of the cavity‐type sensor with the silica thicknesses ranging from 0 to 150 nm. The top layer is an array of close‐packed Au@Pd@oxide (19/2) NPs. The dark and light dotted lines indicate the reflectance maxima of RC mode and gap mode, respectively. (B) Contour plot of simulated reflection spectra of the cavity‐type sensor after H_2_ exposure. The top layer is an array of slightly fused Au@Pd (19/2) NPs. (C) Experimental results: changes of the reflection spectra of the cavity‐type sensors with various silica thicknesses before and after H_2_ exposure. The silica thicknesses are 19, 58, and 143 nm from top to bottom.

To optimize sensing performance, we systematically investigated the effect of the silica interlayer dimension. Figure 5 demonstrates the evolution of calculated and measured reflection spectra versus silica thickness (0–150 nm) before and after H_2_ exposure. For the pristine sensors, calculated reflectance peaks corresponding to the gap mode can be clearly seen as indicated by the white dotted line in Figure 5A. These reflectance peaks vanish after the slight fusion of the Au@Pd NPs. For relatively thin interlayers (<20 nm), the cavities cannot support any RC mode in the visible light range. In this case, their optical properties are dominated by the near‐field coupling between the top NPs and the bottom Ag mirror. Therefore, the H_2_‐induced PdO reduction has little influence on their far‐field spectra (Figure 5C). At larger interlayer thicknesses (>70 nm), both the RC mode (dark dotted line in Figure 5A) and the broad gap mode can be observed in the visible range. As the silica thickness increases, the RC mode redshifts and spectrally overlaps with the gap mode. For example, when the silica thickness reaches 143 nm, the reflection spectrum is governed by the H_2_‐insensitive RC mode with much higher reflectance (Figure 5C). Consequently, the overlap between these two modes leads to an imperceptible color change.

Based on the above analysis, we establish that optimal performance requires satisfying the following three criteria simultaneously: spectral separation of RC and gap modes to preserve distinguishable features, comparable intensity between the two modes in the visible range to maximize contrast, and overlap of the gap mode with the absorption background to ensure complete quenching upon hydrogen exposure. The ∼58 nm silica thickness in our experiments simultaneously fulfills these requirements, thus maximizing color discrimination while maintaining insensitivity to viewing angle and background. This systematic thickness optimization, validated by experiment‐simulation consistency, not only enhances sensor performance but also provides general design principles for future eye‐readable gas sensors.

## 3 Conclusion

In summary, we have developed a FP cavity‐structured EHS based on a novel HICT mechanism. This approach achieves unprecedented performance: ultralow CDC (0.1% H_2_ in air), rapid response (4 s at ≥2% H_2_), and exceptional reliability with background‐, angle‐, and humidity‐insensitive operation. HICT mechanism fundamentally differs from conventional dielectric constant change or volume expansion resulting from the Pd‐PdH*_x_* transition. Both experimental and computational results confirm that the dramatic optical response stems from an active plasmonic mode transition from gap mode to CT mode, triggered by H_2_‐induced reduction of the PdO shell. This HICT mechanism promises a rapid response rate as the kinetics of PdO reduction can be catalytically accelerated by Pd. The FP cavity architecture further eliminates background interference and enhances colorimetric contrast by providing a low‐noise optical background. Combined with the scalable fabrication strategy, these advances enable real‐world deployment for visual hydrogen leak detection. As demonstrated, the sensor rapidly and visibly localizes hydrogen leak points on curved pipeline surfaces through an instantaneous color transition without the need for external instrumentation. To the best of our knowledge, this work represents the first experimental and theoretical demonstration of actively switched plasmon modes for EHS applications. We believe this study establishes a whole new paradigm for the design of next‐generation colorimetric sensors, offering a robust pathway toward safe, real‐time hydrogen monitoring in industrial and energy infrastructure applications.

## 4 Experimental Section

### 4.1 Materials

All glassware was washed using aqua regia (concentrate HCl/ concentrate HNO_3_ = 3:1) and then soaked in a piranha solution (concentrate H_2_SO_4_/30% H_2_O_2_ = 7:3) for 1 h. Afterward, they were repeatedly rinsed with deionized water (18.2 MΩ cm). All chemical reagents were used without any additional purification.

### 4.2 Synthesis of Au@Pd NPs

First, Au sol (∼19 nm) was prepared according to the previously reported method [49]. In brief, 1.21 mL of HAuCl_4_ solution (1%) was mixed with 100 mL of deionized water and boiled. After that, 2.5 mL of sodium citrate (1%) was added into the system, which was boiled for another 20 min and then cooled down. Then, Au@Pd NPs were prepared by a seed‐growth method with Au NPs as the seeds. Briefly, 138 µL of H_2_PdCl_4_ (54.6 mM) was first added into 30 mL of 19 nm Au sol, and then, a mixture containing 215 µL of sodium citrate (1%), 430 µL of ascorbic acid (1%), and 9.35 mL of water was added dropwise into the solution within 30 min under stirring. The overall thickness of the Pd shell with the native oxide is ∼2 nm.

### 4.3 Preparation of Close‐Packed NPs

Close‐packed Au@Pd@oxide NPs were prepared using a three‐phase self‐assembly process with Marangoni compression effect as described previously [28]. Briefly, 4 mL of Au@Pd sol was added into a centrifuge tube (10 mL), and then, 2 mL of CH_2_Cl_2_ was added to form a water/CH_2_Cl_2_ interface, then moderately shake the centrifuge tube for one minute. After that, 800 µL of n‐hexane was added into the centrifuge tube, and Au@Pd NPs were spontaneously assembled into Au@Pd NPs monolayer at the water/n‐hexane interface. Subsequently, the n‐hexane was removed, and the Au@Pd NPs can be transferred onto a solid substrate by a lift‐up method. Once these NPs were exposed to air, an oxide layer would grow quickly on the NP surface due to the high activity of Pd, and thus described as Au@Pd@oxide NPs.

### 4.4 Preparation of the Cavity‐Type Sensor

To prepare a FP cavity‐type sensor, a thin Ag film (∼30 nm) was first coated on a glass slide using high‐vacuum thermal evaporation. Then, a silica interlayer was prepared by spin‐coating silica precursor onto the Ag film and heating at 135°C for 10 min. The silica precursor was synthesized according to the previously reported method [50]. Briefly, 5 mL of tetraethoxysilane (TEOS) was mixed with 5 mL of ethanol. The resulting solution was added dropwise over 20 min into a mixture containing 1.935 mL of deionized water and 81 µL of concentrated hydrochloric acid maintained at 70°C. The reaction mixture was stirred at 70°C for 2 h, cooled to room temperature, and further stirred for 24 h. The thickness of the silica interlayer can be tuned by adjusting the precursor concentration (Figure S10). For example, when diluted with ethanol with a 1:7 ratio, the obtained silica interlayer thickness is ∼58 nm. Finally, the pre‐assembled monolayer of close‐packed Au@Pd@oxide NPs was transferred onto the silica interlayer. The FP cavity‐type sensor was also prepared on a flexible PET substrate in order to adhere to the curved surface of a hydrogen gas pipeline.

### 4.5 Characterization of the Sensing Performance of the As‐Prepared Sensors

A device consisting of a spectrometer (Maya 2000PRO, Ocean Optics), a light source (DH‐2000‐BAL, Ocean Optics), and a 2 × 1 fiber optic coupler was used to measure the reflection of the as‐prepared sensors in the visible light region. The sample was placed in a silica circulating cuvette (1 × 1 × 3.7 cm^3^) (Figure S11). When the measurement started, the circulating cuvette was connected with a H_2_/synthetic air mixture, with gas flow rate of 10 mL·s^−1^. The optical property of the sample could be recorded in real‐time when hydrogen gas was introduced. All of the optical measurements were performed at room temperature.

### 4.6 FDTD Calculations

We calculated the optical properties of the monolayer‐type and cavity‐type sensor using the Ansys Lumerical FDTD 3D electromagnetic solver. Concentric core‐shell spheres were built as the models of Au@Pd@oxide and Au@Pd NPs, where the sizes were set according to the TEM measurements. The thickness of the oxide shell was set as 0.5 nm. The permittivity of Ag, Pd, and silica were modeled according to the *Handbook of Optical Constants of Solids* by Palik [51] and the permittivity of Au was adopted from John and Christy [52]. The refractive index of PdO was set as 3.3 according to the experimental data by Nilsson et al. [53]. The dielectric constant of air was set as 1. A plane wave from 400 to 1050 nm was used as the light source with a normal incidence. The NP monolayer was enclosed by a fine mesh box with the maximum mesh step of 0.2 nm. The optimization of silica thickness was performed by running a parameter sweep using the built‐in sweep utility. The thickness of silica was swept from 0 to 150 nm with an interval of 2.5 nm.

### 4.7 Characterization

A Hitachi SU1080 field emission SEM was used to analyze the morphology of the Au@Pd NPs at a primary electron energy of 15 kV. TEM images were taken by a JEOL 2100F electron microscope with a primary electron energy of 200 kV. XPS spectra were measured in the vacuum condition by a THERMOVG ESCALAB 250 (Thermo VG) setup with Al Kα x‐rays.

## Author Contributions

X. Z., B. W. and H. Zeng designed the project. X. Z. and S. Y. led the paper writing. L. C., C. L., Z. C. and T. W. performed the experiments and carried out the data analysis. L. C., C. L. and J. Z. drafted the Figures. S.Y. performed FDTD calculation. F. L. revised the manuscript. B. W. and H. Zeng supervised the project and revised the manuscript. All authors contributed to discussions of the results.

## Funding

This work was supported by the National Natural Science Foundation of China (22175031, 22305030, 52301115, 22075039).

## Conflicts of Interest

The authors declare no conflicts of interest.

## Supporting information




**Supporting File 1**: advs77338‐sup‐0001‐SuppMat.docx.

**Supporting File 2**: advs77338‐sup‐0002‐S1.mp4.

## Data Availability

The data that support the findings of this study are available from the corresponding author upon reasonable request.
