# Supplementary material for: Cavity‐Enhanced Eye‐Readable Detection of 0.1% H2 via Active Modulation of Charge‐Transfer Plasmons
Source: Adv Sci (Weinh). 2026 Aug 27;13(61):e77338. doi: 10.1002/advs.77338 (PMC13521180; doi:10.1002/advs.77338)
Supplement: Supplementary file 1 [file ADVS-13-e77338-s001.docx]

**Supporting Information for**

**Cavity-Enhanced Eye-Readable Detection of 0.1% H_2_ via Active Modulation of Charge-Transfer Plasmons**

Xuemin Zhang^*^, Shunsheng Ye, Lubing Cai, Chao Li, Zheng Cai, Jin Zhang, Fei Liu, Tieqiang Wang, Boxin Wei^*^, Hongbo Zeng^*^

X. Zhang, S. Ye, L. Cai, C. Li, T. Wang

Department of Chemistry, College of Sciences, Northeastern University, Shenyang, Liaoning 110819, P. R. China

E-mail: zhangxuemin@mail.neu.edu.cn (X. Zhang)

Z. Cai

Institute of Metal Research, Chinese Academy of Sciences, Shenyang 110016, P. R. China

F. Liu

National & Local Joint Engineering Laboratory for High-Performance Metal Structure Material and Advanced Manufacturing Technology, College of Materials and Metallurgy, Guizhou University, Guiyang, Guizhou 550025, P. R. China

B. Wei

Institute of Metal Research, Chinese Academy of Sciences, Shenyang 110016, P. R. China;

School of Mechanical and Aerospace Engineering, Nanyang Technological University, Singapore, Singapore

E-mail: bxwei17s@imr.ac.cn (Boxin Wei)

J. Zhang, H. Zeng

Department of Chemical and Materials Engineering, University of Alberta, Edmonton, T6G 1H9, Canada

E-mail: hongbo.zeng@ualberta.ca (Hongbo Zeng)

**Figure S1.** XRD pattern of Au@Pd@oxide NPs before and after H_2_ exposure

**Figure S2.** Optical responses of the HICT-based sensors to pure H_2_. (A) Au@Pd@oxide (19/4) NPs, (B) Au@Pd@oxide (35/2) NPs, (C) pure Au NPs and (D) pure Pd NPs were assembled into close-packed monolayers and used as the top layers of FP cavity-type sensors.

**Figure S3.** Definitions of sensor response for the cavity-type sensor (A) and monolayer-type sensor (B). The sensor response *S* is defined as (*I*_a_ - *I*_g_)/ *I*_a_, where *I*_a_ and *I*_g_ are the maximum reflectance of the sensor in air and H_2_, respectively.

**Figure S4** Performance of as prepared sensor in 4% H_2_ (mixed with 30% CH_4_), and 4% H_2_ pre-exposed to other interference gases.

**Figure S5** Sensing performance of commercial hydrogen tape in 0.2%, 1% and 4% H_2_.

**Figure S6** Photograph (A) and hydrogen sensing response (B) of the as-prepared sensor fabricated on a PET substrate in the bent state.

**Figure S7** SEM images of the Au@Pd@oxide (19/2) NP array (A) before and (B) after exposure to 100% H_2_. The two images were taken at exactly the same location on the sample.

**Figure S8** Optical responses of the Au@Pd@oxide (19/2) monolayer-type sensor towards (A) 100% H_2_ and (B) 0.3% H_2_.

**Figure S9** (A) A schematic illustration of the Au@Pd@oxide (19/2) monolayer-type sensor. (B) Hydrogen-induced discoloration of the Au@Pd@oxide (19/2) monolayer-type sensor against light and dark backgrounds. (C) Calculated reflection, transmission, and absorption spectra of the monolayer-type sensor. (D) Responses of the monolayer-type and cavity-type sensors to pure H_2_ as functions of time.

**Figure S10** Silica thickness plotted as a function of the dilution rate of the original silica precursor. The precursor solution was diluted with ethanol.

**Figure 11** Optical characterization system for real-time monitoring of reflection spectrum. The sensor is placed inside a transparent, sealed gas-flow cuvette. The gas flow rate is ~10 mL·s^-1^.

**Table S1.** **Summary of the sensing performance of state-of-the-art plasmonic EHS systems**

| Structure | CDC | Response time  (*t*_90_) | Background | Mechanism | Ref. |
| --- | --- | --- | --- | --- | --- |
| Au@Pd octahedras | 100% (from purplish red to purple) | ~20 s  (100% H_2_) | Water | HIDC | ^[1]^ |
| Au@Pd nanorods | 2% (from brightness to darkness) | ~240 s  (2% H_2_) | N_2_ | HIVE | ^[2]^ |
| Pd/PUA nanofibers | 4% (from low transparency to high transparency) | ~5.1 s  (4% H_2_) | N_2_ | HIVE | ^[3]^ |
| Pd/Cr/Ti micro-rolls | 1% (from transparent to opaque) | ~4.7 s  (4% H_2_) | N_2_ | HIVE | ^[4]^ |
| Pd film on PDMS | 0.6% (from gray to brightness) | ~7 s  (4% H_2_) | N_2_ | HIVE | ^[5]^ |
| Au/Pd film on PDMS | 4% (from flat to wrinkled) | ~50 s  (4% H_2_) | N_2_ | HIVE | ^[6]^ |
| Au@Pd NPs array | 1% (from turquoise to gray) | ~225 s  (4% H_2_) | Air | HIVE | ^[7]^ |
| Au@Pt NPs array | 10% (from turquoise to gray) | ~225 s  (10% H_2_) | Air | HIVE | ^[8]^ |
| Pd/Au cavity | 0.4% (from red to lime green) | ~9 s  (0.6% H_2_) | Air | HIVE | ^[9]^ |
| Pd NPs-based cavity | <4% (from pink to black) | ~27 s  (4% H_2_) | Air | HIVE | ^[10]^ |
| **This work** | **0.1% (from pink to blue)** | **~4 s**  **(4% H_2_)** | **Air** | **HICT** |  |

**Table S2.** **Summary of the sensing performance of other-type EHS systems**

| Material | CDC | Response time (*t*_90_) | Δ*E* | Coloring rate*  [Δ*E*/(*t*_90_×*C*_H2_)] | Reference |
| --- | --- | --- | --- | --- | --- |
| Pd-WO_3_ nanocomposites | <1% | ~200 s  (1% H_2_) | ~10  (1% H_2_) | ~5 s^-1^ | ^[11]^ |
| Pd-WO_3_ film | <1% | ~200 s  (1% H_2_) | ~60  (1% H_2_) | ~30 s^-1^ | ^[12]^ |
| Pd-Au@PdO  NPs | 1% | ~20 min  (1% H_2_) | ~8  (1% H_2_) | ~0.7 s^-1^ | ^[13]^ |
| PdO-ZnO  NPs | 1% | ~100 s  (4% H_2_) | ~65  (4% H_2_) | ~16.3 s^-1^ | ^[14]^ |
| Pd-MO_3_ nanocomposites | 0.6% | ~1000 s  (1 % H_2_) | ~177  (1 % H_2_) | ~17.7 s^-1^ | ^[15]^ |
| Commercial H_2_ detection tape** | 1% | ~300 s  (1% H_2_) | ~7  (1% H_2_) | ~2.3 s^-1^ |  |
| **This work** | **0.1%** | **~4 s**  **(4% H_2_)** | **~31**  (4% H_2_) | **~193.8** s^-1^ |  |

*****Coloring rate is calculated based on the data in corresponding references.

**Yinshi Technology, China.

[1]	C. Y. Chiu, M. H. Huang, *Angew. Chem. Int. Ed.* **2013**, *52*, 12709-12713.

[2]	M. E. Nasir, W. Dickson, G. A. Wurtz, W. P. Wardley, A. V. Zayats, *Adv. Mater.* **2014**, *26*, 3532-3537.

[3]	H. Han, S. Baik, B. Xu, J. Seo, S. Lee, S. Shin, J. Lee, J. H. Koo, Y. Mei, C. Pang, T. Lee, *Adv. Funct. Mater.* **2017**, *27*, 1701618.

[4]	B. Xu, Z. Tian, J. Wang, H. Han, T. Lee, Y. Mei, *Sci. Adv.* **2018**, *4*, eaap8203.

[5]	X. She, Y. Shen, J. Wang, C. Jin, *Light Sci Appl* **2019**, *8*, 4.

[6]	X. She, H. Du, Y. Shen, N. X. Fang**,** C. Jin**,** *ACS Appl. Mater. Interfaces* **2022**, *14*, 41531–41541.

[7]	C. Li, H. Zhu, Y. Guo, S. Ye, T. Wang, Y. Fu, X. Zhang, *ACS Sens.* **2022**, *7*, 2778-2787.

[8]	X. She, Q. Yao, Q. Zou, G. Yang, Y. Shen, C. Jin, *ACS Appl. Mater. Interfaces* **2023**, *15*, 16244-16252.

[9]	P. Ren, S. Ye, C. Li, L. Cai, F. Zheng, T. Wang, B. Wei, X. Zhang, *ACS Sens.* **2025**, *10*, 8098-8108.

[10]	Z. Liu, C. Wang, Y. Yang, N. Zhang, Y. Shen, X. She, C. Jin, *ACS Sens.* **2025**, *10*, 6859-6868.

[11] R. Yeasmin, G. Jung, S. Han, C. Park, H. Seo, *Chem. Eng. J.* **2024**, *482*, 148911.

[12] S. H. Cho, J. M. Suh, B. Jeong, T. H. Lee, K. S. Choi, T. H. Eom, T. Kim, H. W. Jang, *Chem. Eng. J.* **2022**, *446*, 136862.

[13] G. Chen, C. Li, L. Lu, P. Li, S. Ye, T. Wang, Y. Fu, X. Zhang, *Sens. Actuators B Chem.* **2023**, *393*, 134199.

[14] Y. K. Kim, S.-H. Hwang, S. M. Jeong, K. Y. Son, S. K. Lim, *Talanta* **2018**, *188*, 356-364.

[15] X. Sun, L. Hao, L. Chen, X. Guo, C. Han, J. Chen, W. Jiao, R. Wang, X. He, *Appl. Surf. Sci.* **2022**, *599*, 153878.
